# Genetic Syndromes and Multimorbidity in Adults with Congenital Heart Disease and Heart Failure: Insights from the PATHFINDER-CHD Registry

**DOI:** 10.3390/jcm15031290

**Published:** 2026-02-06

**Authors:** Ann-Sophie Kaemmerer-Suleiman, Fritz Mellert, Stephan Achenbach, Pinar Bambul-Heck, Robert Cesnjevar, Oliver Dewald, Helena Dreher, Andreas Eicken, Anna Engel, Peter Ewert, Annika Freiberger, Jürgen Hörer, Christopher Hohmann, Stefan Holdenrieder, Michael Huntgeburth, Harald Kaemmerer, Renate Kaulitz, Frank Klawonn, Christian Meierhofer, Steffen Montenbruck, Nicole Nagdyman, Rhoia C. Neidenbach, Robert D. Pittrow, Christoph R. Sinning, Fabian von Scheidt, Pelagija Zlatic, Frank Harig, Mathieu N. Suleiman

**Affiliations:** 1 Department of Cardiac Surgery, University Hospital Erlangen, Friedrich-Alexander-University Erlangen-Nürnberg, 91054 Erlangen, Germany; 2 Department of Cardiology, University Hospital Erlangen, Friedrich-Alexander-University Erlangen-Nürnberg, 91054 Erlangen, Germany; 3 International Center for Adults with Congenital Heart Disease, Department of Congenital Heart Disease and Paediatric Cardiology, TUM University Hospital, German Heart Center Munich, 80636 Munich, Germany; 4 Klinik für Herz-und Gefäßchirurgie am Klinikum Karlsburg, Herz-und Diabeteszentrum MV, 17495 Karlsburg, Germany; 5 Department of Congenital and Paediatric Heart Surgery, German Heart Center Munich, University Hospital of the Technical University, 80636 Munich, Germany; 6 Division of Congenital and Pediatric Heart Surgery, European Kids Heart Center, University Hospital Großhadern, Ludwig-Maximilians-University Munich (LMU), 81377 Munich, Germany; 7 Clinic III for Internal Medicine, Heart Center, Faculty of Medicine, University Hospital of Cologne, University of Cologne, 50937 Cologne, Germany; 8 Department of Laboratory Medicine, German Heart Centre Munich, Technical University of Munich, 80636 Munich, Germany; 9 Pediatric Cardiology, Universitätsklinikum Tübingen, 72076 Tübingen, Germany; 10 Helmholtz Centre for Infection Research, Biostatistics, Technical University Braunschweig, 38124 Braunschweig, Germany; 11 Department of Sport and Human Movement Science, University of Vienna, 1010 Vienna, Austria; 12 Department of Cardiology, University Heart and Vascular Centre Hamburg, 20246 Hamburg, Germany

**Keywords:** adult congenital heart disease, genetic syndrome, aging, PATHFINDER-CHD Registry, registry, heart failure

## Abstract

**Background/Objectives:** Progress in diagnostic and therapeutic strategies has resulted in an increasing prevalence of adults with congenital heart disease (ACHD), including those involving genetically determined syndromes. This study aimed to characterize prevalence, congenital phenotypes, heart failure (HF) stages, comorbidity burden, and current medical management of ACHD and concomitant genetically determined syndromes enrolled in a prospective HF-focused registry. **Methods:** The PATHFINDER-CHD Registry is a German-based (est. 2022) multicenter observational registry. This web-based platform consecutively tracks ACHD patients across the heart failure spectrum, including those with current or prior HF, as well as those at high structural or functional risk. HF stage was classified using a modified ACC/AHA scheme adapted for CHD; functional capacity was graded according to the Perloff classification. Baseline demographics, CHD anatomy, prior surgical/interventional treatment, cardiac and extracardiac comorbidities, and medication were collected from medical records. **Results**: Among 1987 enrolled ACHD, 107 (5.4%) had a genetic syndrome (n = 65, 60.7% women; mean age 33.5 ± 9.4 years; range 18–68). Most common syndromes were trisomy 21 (n = 49; 45.8%) and 22q11.2 deletion (n = 27; 25.2%); 31 patients (30.0) had rarer syndromes. Predominant CHD diagnoses were atrioventricular septal defect (n = 42, 39.3%), tetralogy of Fallot (n = 19, 17.8%), and pulmonary atresia with ventricular septal defect (n = 7, 6.5%). A systemic left ventricle was present in 102 (95.3%); 40 (37.4%) had primarily cyanotic CHD, and 7 (6.5%) an Eisenmenger physiology. Most patients (n = 71; 66.4%) had undergone definite surgical repair; 25 patients (23.3%) had at least one catheter intervention, including transcatheter valve implantation in 17 cases (15.9%). HF stage was mainly B (n = 30, 28.0%) or C (n = 75, 70.1%). Perloff functional class I/II was present in 97 (90.7%). Leading cardiac comorbidities included intrinsic aortopathy (n = 49, 45.8%), pulmonary arterial hypertension (n = 12, 11.2%), and arrhythmias (n = 10, 9.3%). Frequent extracardiac comorbidities were thyroid dysfunction (n = 34, 31.8%), kidney disease (n = 16, 15.0%), hyperuricemia (n = 13, 12.1%), and depression (n = 15, 14.0%). Pharmacotherapy was used in 66 patients (61.7%). Beta-blockers (n = 25, 23.4%) were common, while ACEi/ARB (n = 9, 8.4%), diuretics (n = 10, 9.3%), MRAs (n = 8, 7.5%), and SGLT2 inhibitors (n = 3; 2.8%) were infrequently prescribed; no patient received ARNI or digitalis. For targeted treatment of pulmonary arterial hypertension, phosphodiesterase-5 inhibitors (n = 7, 6.5%), endothelin receptor antagonists (n = 6, 5.6%), or prostacyclin analogues (n = 1, 0.9%) were used. As oral anticoagulants, vitamin K antagonists or direct oral anticoagulants (DOACs) were prescribed in 17 cases (15.9%). Forty-one patients (38.3%) received thyroid hormone replacement. **Conclusions:** Syndromic ACHD constitute a small but clinically high-risk subgroup within an HF-oriented registry, marked by complex CHD, substantial cardio–extracardiac multimorbidity (notably aortopathy, PAH, thyroid disease, renal dysfunction, depression), and low utilization of contemporary HF therapies. These data support specialized, interdisciplinary, longitudinal care pathways and prospective studies addressing outcomes and evidence-based HF management in syndromic ACHD.

## 1. Introduction

Decades of innovation in congenital cardiac care—from advanced diagnostics to specialized surgery and perioperative support—have drastically improved the life expectancy and quality of life for the CHD population. In contemporary industrialized nations, the survival rate for children born with congenital heart disease (CHD) now exceeds 95%, shifting the clinical focus toward long-term adult care. Moreover, adults with congenital heart disease (ACHD) are meanwhile not only reaching middle age, but an increasing proportion are also entering their sixth and seventh decades of life [1,2]. This demographic transition has created a novel and heterogeneous cohort of older ACHD characterized by complex residua and sequelae, acquired comorbidities, and unique age-related challenges.

Within this expanding population, a substantial subset presents with underlying genetic syndromes, such as trisomy 21 (Down syndrome), 22q11.2 deletion syndrome, Turner syndrome, Noonan syndrome, Williams–Beuren syndrome, and even rarer syndromes, reflecting the fundamental contribution of genetic and developmental mechanisms to congenital cardiac morphogenesis.

As survival into older age increases, the long-term implications of these syndromes are becoming increasingly apparent. They range from multisystem involvement and neurocognitive disorders to endocrine, metabolic, and psychiatric comorbidities.

While surgical and interventional outcomes have improved considerably also in syndromic CHD, adult and geriatric data remain limited. Recent registry and cohort studies suggest that syndromic ACHD experience accelerated biological aging, higher multimorbidity burdens, and distinct patterns of heart failure, pulmonary vascular disease, arrhythmia, and aortic complications compared with non-syndromic peers.

Heart failure in ACHD differs from acquired HF in that it is strongly influenced by congenital anatomy, prior surgical/interventional repair, and lesion-specific physiology. In particular, systemic right ventricular function, univentricular/Fontan circulation, residual hemodynamic lesions, and pulmonary vascular disease may shape HF presentation, treatment tolerance, and prognosis. Moreover, ACHD, especially syndromic ACHD, often exhibits substantial cardiac and extracardiac multimorbidity, which can complicate pharmacological management (e.g., blood pressure reserve, renal/hepatic vulnerability, polypharmacy) and contributes to care complexity. Together with the underrepresentation of ACHD in pivotal HF trials, this creates a relevant evidence gap regarding contemporary HF management in syndromic ACHD.

Heart failure (HF) is the leading cause of illness and death in ACHD, including those with genetic syndromes, and often results from a combination of the underlying genetic factors, abnormal cardiac anatomy, prior surgeries, and lifelong hemodynamic stress. Despite these challenges, current ACHD literature addresses genetic syndromes only marginally, and evidence-based care models for aging syndromic adults with CHD remain scarce [3,4,5,6,7].

The present work aims to characterize genetic syndromes among aging ACHD, describe their management as well as cardiovascular and extracardiac comorbidities, and highlight the implications for long-term management, functional capacity, and health care structures. Seeking to close this knowledge gap, our study focused on syndromic patients consecutively enrolled in the PATHFINDER-CHD Registry. This web-based, prospective platform tracks ACHD across the heart failure spectrum, including those with prior HF or high structural risk [8,9].

By combining registry data with focused literature evidence, this study seeks to highlight specific challenges such as the need for age-adapted care models to fill a critical knowledge gap at the interface of congenital cardiology, genetics, and preventive and aging medicine.

## 2. Materials and Methods

### 2.1. Study Design and Objectives

This study aimed to explore the bidirectional relationship between syndromic/genetically determined disorders and aging in adults with congenital heart disease (ACHD), and to assess their association with clinical phenotype, cardiac and extracardiac comorbidities, and contemporary management patterns.

### 2.2. Registry and Study Population

Established in 2022, the PATHFINDER-CHD Registry is a German prospective, multicenter, web-based platform designed to systematically track heart failure in ACHD. Inclusion criteria require a diagnosis of ACHD alongside manifest HF, a prior history of HF, or high structural/functional risk for HF [8].

Participating centers include the International Center for Adult Congenital Heart Disease at the German Heart Center Munich, the Departments of Cardiac Surgery and Cardiology at Friedrich-Alexander-University Erlangen-Nürnberg, the Department of Pediatric Cardiology at University Hospital Tübingen, and the Department of Cardiology at the University of Cologne and Hamburg.

Patients were enrolled consecutively and without preselection during routine outpatient visits or hospital admissions at participating institutions.

### 2.3. Definition of Syndromic and Genetically Determined Disorders

For the present analysis, we used the generic term “genetic syndromes/disorders” and differentiated between two categories, (1) classical genetic syndromes (chromosomal or monogenic conditions typically associated with dysmorphic and/or neurodevelopmental features) and (2) other genetically determined disorders with potential relevance for ACHD management (e.g., inherited bleeding disorders such as von Willebrand disease), as the PATHFINDER-CHD Registry is designed to capture genetic comorbidities that may modify clinical management, risk stratification, and outcomes in ACHD. Diagnoses were based on documented clinical diagnoses and/or available genetic testing results in the medical records.

### 2.4. Heart Failure Staging and Functional Classification

HF stage was assigned according to the ACC/AHA stage definitions based on chart-documented structural congenital heart disease and the presence/absence of current or prior HF symptoms (Stage B: structural disease without HF symptoms; Stage C: structural disease with current/prior HF symptoms; Stage D: advanced HF with persistent symptoms despite medical therapy). Stage A was not applicable because all registry participants have structural CHD by definition [10,11].

Functional status was assessed using Perloff classification, which categorizes ACHD patients according to symptom-related limitation in daily life [1,12].

A systematic review of medical records was performed to obtain physician-documented diagnoses for all demographic and clinical variables. Data were captured using a structured case report form and included age, sex, CHD diagnosis, and HF classification. Anthropometric measurements, oxygen saturation, medication use, and a history of surgical or interventional treatments were also recorded.

### 2.5. Ethics/Consent/Declaration of Helsinki

In accordance with the Declaration of Helsinki, the registry received ethical clearance from all participating sites, including the Technical University Munich (Reference Nr: 158/19S) and the Friedrich-Alexander-University Erlangen-Nürnberg (Reference Nr: 22-56-Bn). Compliance with Good Pharmacoepidemiological Practice (GPP) and data protection guidelines was maintained throughout. All subjects provided written informed consent for their participation.

### 2.6. Statistical Analysis

Data analysis was performed using R version 4.4.3 on pseudonymized datasets. Descriptive statistics for numeric variables include the mean ± SD, median, and range. To assess differences between groups, we employed the Wilcoxon rank-sum test for continuous data and either Pearson’s Chi-squared, Fisher’s exact, or z-tests for categorical proportions. A two-sided *p*-value of <0.05 was considered statistically significant.

We analyzed the available data for each variable and report the corresponding denominators. No imputation was performed.

### 2.7. Data Collection

Patient demographics and clinical characteristics were extracted via systematic medical record review and documented using a standardized case report form. The dataset encompassed age, sex, primary CHD anatomy, and heart failure (HF) stage. Additionally, we recorded a comprehensive range of comorbidities, anthropometric measurements (height, weight, and BMI), and resting oxygen saturation. Treatment history was captured through detailed documentation of current pharmacotherapy and prior surgical or catheter-based interventions.

### 2.8. Exclusion Criteria

Patients were excluded if they were unable to provide informed consent or declined participation.

## 3. Results

Among 1987 adults with congenital heart disease (ACHD) enrolled up to December 2025 in the PATHFINDER-CHD Registry, 107 (5.4%) had a concomitant genetically determined syndrome or disorder and were included in the present analysis. The cohort comprised 65 women (60.7%) and 42 men (39.3%).

Trisomy 21 was present in 49 patients (45.8%), 22q11.2 deletion syndrome in 27 (25.2%), Ullrich–Turner syndrome in 4 (3.7%), and Noonan syndrome in 4 (3.7%). The remaining 23 patients (21.5%) had rarer diagnoses, such as 4q deletion syndrome with psychomotor retardation, 9q34.3 deletion, Asperger syndrome, Cornelia de Lange syndrome, 18q deletion, familial facial nerve (VII) palsy syndrome, Gitelman or Bartter syndrome, Goldenhar syndrome, Holt–Oram or Levy–Hollister syndrome, homozygous MTHFR mutation, Klippel–Feil syndrome, complex malformation syndrome, Mayer-Rokitansky-Küster-Hauser syndrome, multiorgan dysfunction syndrome, multiple dysmorphic features syndrome, Bardet–Biedl syndrome, orofaciodigital syndrome, Prader–Willi syndrome, von Willebrand–Jürgens syndrome, and Williams–Beuren syndrome.

### 3.1. Demographic Data

The mean age was 33.4 years, without a significant sex difference. Mean height was 157 cm and mean body weight was 68 kg, corresponding to a mean body mass index (BMI) of 28 kg/m^2^. The patients exhibited notably short stature, potentially attributable to the underlying genetic syndromes (Table 1).

### 3.2. Congenital Heart Disease

The cohort encompassed a broad spectrum of repaired or native CHD, including conotruncal defects, shunt lesions, and right- and left-sided anomalies. Atrioventricular septal defect (n = 42, 39.3%) and tetralogy of Fallot (n = 19, 17.8%) constituted the majority of the underlying diagnoses. These were followed by pulmonary atresia with ventricular septal defect (n = 7, 6.5%) and isolated ventricular septal defect (n = 6, 5.6%) (Table 2).

In terms of ventricular morphology, the vast majority of patients featured a systemic left ventricle (n = 102, 95.3%). Morphologic systemic right ventricle was present in 3 patients (2.8%), while 2 (1.9%) had univentricular circulation (Table 3).

The majority primarily had acyanotic CHD (n = 67, 62.6%), while 40 (37.4%) had primarily cyanotic CHD. Seven patients (6.5%) had secondary cyanosis related to a primary left-to-right shunt lesion (Eisenmenger syndrome) (Table 3).

Regarding prior treatment, 8 patients (7.5%) were treatment-naïve. A total of 71 (66.4%) had undergone definitive reparative cardiac surgery, whereas 3 (2.8%) had undergone only palliative procedures (e.g., pulmonary artery banding or systemic-to-pulmonary shunt placement). The most frequent surgical procedures were ToF repair (n = 23, 21.5%) and heart valve replacement (n = 25, 23.4%). An arterial switch operation, a Rastelli-type operation, and a Ross operation were each performed only once.

Twenty-five patients (23.4%) had undergone at least one catheter-based intervention, either as primary treatment (n = 1) or after previous cardiac surgery (n = 24). Interventional treatment included 17 cases (15.9%) of transcatheter heart valve.

### 3.3. Heart Failure (HF)

All included patients had manifest heart failure (HF) or were considered at risk for HF based on the underlying CHD.

Utilizing the CHD-adapted ACC/AHA staging system, the majority of the cohort was classified as stage C (n = 75, 70.1%) or stage B (n = 30, 28.0%), while only 2 patients (1.9%) were categorized as stage D. Per the study’s inclusion criteria, stage A patients were excluded by definition (Table 4)

Functional capacity was predominantly preserved as 97 patients (90.7%) were in Perloff functional class I/II, 9 (8.4%) in class III, and 1 (0.9%) in class IV (Table 4).

### 3.4. Cardiac and Non-Cardiac Comorbidities

Cardiac comorbidities were dominated by intrinsic aortopathy (n = 49, 45.8%), pulmonary arterial hypertension (n = 12, 11.2%), and clinically relevant arrhythmias (n = 10, 9.3%). Notably, pulmonary arterial hypertension tended to be more prevalent in men than in women (16.7% vs. 7.7%; *p* = 0.262). A history of infective endocarditis was documented in 9 patients (8.4%) (Table 5).

The extracardiac profile of the cohort was characterized by a high prevalence of thyroid dysfunction (n = 34, 31.8%). Other significant comorbidities included renal impairment (n = 16, 15.0%), depressive disorders (n = 15, 14.0%), and hyperuricemia (n = 13, 12.1%) (Table 5).

### 3.5. Pharmacological Treatment

Overall, 66 patients (61.7%) received pharmacological therapy (Table 6). The most prescribed cardiovascular medications were beta-blockers (n = 25/107, 23.4%), ACE inhibitors and/or angiotensin receptor blockers (ACEI/ARBs; n = 9/107, 8.4%), diuretics (n = 10/107, 9.3%), and mineralocorticoid receptor antagonists (MRAs; n = 8/107, 7.5%) (Table 6).

Sodium–glucose cotransporter 2 (SGLT2) inhibitors were used in only 3/107 patients (2.8%). None of the patients received digitalis glycosides or angiotensin receptor–neprilysin inhibitors (ARNIs).

Targeted pulmonary arterial hypertension therapy was administered as mono- or combination therapy, including phosphodiesterase-5 inhibitors (n = 7/107, 6.5%), endothelin receptor antagonists (n = 6/107, 5.6%), and prostacyclin analogues (n = 1/107, 0.9%).

Overall, 18.7% of patients (n = 20/107) received antithrombotic treatment. The distribution consisted of vitamin K antagonists (n = 14), followed by antiplatelet therapy (n = 4) and DOACs (n = 3).

Thyroid hormone replacement therapy was used in 41/107 patients (38.3%), and 6 (5.6%) received oral iron supplementation (Table 6).

## 4. Discussion

In this analysis of the PATHFINDER-CHD Registry, adults with congenital heart disease (ACHD) and a concomitant genetically determined syndrome were systematically characterized [8]. Among 1987 patients (1051 men, 936 women), 107 (5.4%) had an additional syndrome or genetically determined disorder. However, this represents a small subgroup, which is clinically highly relevant because of concomitant CHD, substantial cardiac and non-cardiac multimorbidity, and low utilization of contemporary HF therapies.

This report complements prior PATHFINDER-CHD publications on broader registry topics, such as aging, obesity, or cachexia, by specifically focusing on the syndromic/genetic subgroup. It provides a structured characterization of their multimorbidity profile and HF-related management in contemporary routine care.

The broad spectrum of syndromes and genetic disorders observed in this cohort of ACHD underscores its heterogeneity and highlights the need for multidisciplinary care in close collaboration with clinical genetics and other specialties.

An age up to 68 years and a mean age of 33 ± 9 years illustrate that, today, syndromic ACHD increasingly reach older age. This reflects the advances in congenital cardiology, cardiac surgery, and specialized ACHD care. At the same time, it shifts the focus toward long-term management, requiring not only CHD expertise but also syndrome-specific knowledge.

The genetic spectrum was dominated by trisomy 21 and 22q11.2 deletion syndrome, together accounting for 71% of the cohort. This aligns with established genotype–phenotype associations.

Trisomy 21 is classically linked to atrioventricular septal defects (AVSDs), while 22q11.2 deletion syndrome is predominantly associated with conotruncal anomalies. Reflecting these established associations, AVSDs (n = 42, 39.3%) and conotruncal defects (n = 37, 34.6%) were highly prevalent in our cohort. The latter group included tetralogy of Fallot (ToF), pulmonary atresia with ventricular septal defect (PA-VSD), truncus arteriosus communis (TAC), double-outlet right ventricle (DORV), and transposition of the great arteries (TGA).

Most patients had a morphologically left systemic ventricle (n = 102, 95.3%). Only a minority had a morphologically right systemic ventricle or univentricular circulation. This may reflect the rarity of the combination of syndromic disease and complex CHD and/or limited long-term survival in highly complex CHD, which reduces the likelihood of reaching older age. However, survivorship bias cannot be excluded.

Notably, a substantial proportion had primary cyanotic CHD (n = 40, 37%), including a small but clinically relevant subgroup with Eisenmenger syndrome (n = 7, 6.5%). This indicates that a meaningful fraction of the population was exposed to substantial hemodynamic vascular and pulmonary vascular burden, which increases the risk of heart failure and pulmonary hypertension.

Most patients had undergone prior cardiac surgery, most commonly corrective cardiac repair (n = 95, 88.8%), whereas palliative procedures were rare (5.6%). ToF repair and valve replacement procedures were particularly frequent.

In addition, approximately one quarter of the cohort (n = 25, 23.4%) had undergone at least one cardiac catheter-based intervention, including percutaneous transcatheter valve implantation (n = 17, 15.9%). This reinforces the concept that CHD is a chronic and progressive condition, as residua, sequelae, and progressive valvular dysfunction frequently emerge over time despite successful initial therapy, often necessitating reoperations or catheter-based reinterventions [1,13,14,15].

Importantly, all patients included in this analysis either had established heart failure or were at increased risk of developing it. Symptomatic heart failure (Stage C) was present in more than half of the patients, while advanced therapy-refractory heart failure (Stage D) was rare (n = 2, 1.9%). Moreover, a substantial proportion had CHD-associated early ventricular dysfunction but were asymptomatic (Stage B; n = 30, 28.0%).

Accordingly, more than 90% of the patients were in Perloff functional class I/II and only mildly limited. This Perloff classification is clinically useful for the stratification and longitudinal assessment of functional limitation, exercise capacity, and symptom-related impairment. When applying an anatomic CHD complexity classification (mild/moderate/severe), all degrees of severity were represented [3,16]. However, anatomy alone may not adequately reflect overall disease burden, as outcomes in complex CHD can improve with specialized follow-up, whereas individuals with anatomically “simple” CHD may develop significant organ dysfunction as they age.

This apparent discrepancy between structural complexity and relatively preserved functional capacity may reflect effective long-term care within specialized ACHD centers. However, symptom underrecognition in syndromic patients is a concern. Cognitive limitations may impair symptom reporting, and proxy assessment may be incomplete. Therefore, objective and systematic functional assessment (e.g., laboratory testing, activity monitoring, exercise testing) is crucial to avoid missing latent or “silent” deterioration.

Extracardiac comorbidity burden was high in the studied patients. Intrinsic aortopathy (n = 49, 46%) affected almost half the cohort, emphasizing that aortic disease is a central component of both CHD and several syndromic disorders [17].

Pulmonary arterial hypertension was present in more than one-tenth of patients (n = 12, 11%), representing a key determinant of prognosis and therapeutic strategy [13,15,18]. Clinically relevant arrhythmias and a history of infective endocarditis further highlight the vulnerability of this population.

Among non-cardiac comorbidities, thyroid disorders (n = 34, 31.8%), renal dysfunction, hyperuricemia (n = 29, 27.1%), and psychiatric disorders, including depression (n = 15, 14%), were common [19]. Thyroid dysfunction may be syndrome-related. In conditions with organ maldevelopment (e.g., 22q11.2 deletion), thyroid disease may reflect developmental disturbances, whereas, in trisomy 21 or Turner syndrome, it more commonly reflects autoimmune thyroiditis (e.g., Hashimoto disease). Iatrogenic contributions are also possible, as various medications (e.g., iron, calcium, antiepileptics) may interfere with thyroid hormone absorption or metabolism. The high rate of thyroid hormone replacement therapy (n = 41, 38.3%) underscores the clinical relevance of endocrinological management.

Depression and other psychiatric disorders are unsurprising given chronic heart disease, syndromic conditions, and reduced autonomy. However, these issues remain underrecognized in routine care. Our findings support systematic screening and integrated mental health pathways as part of long-term follow-up in syndromic ACHD [20].

Pharmacotherapy represents another central aspect of care. While most patients received medical treatment, guideline-directed use of heart failure therapies appeared low. Although conventional heart failure drug classes, including ACE inhibitors/ARBs (n = 9, 8.4%), beta-blockers (n = 25, 23.4%), and diuretics and/or MRAs (n = 18, 16.8%), were used, contemporary regimens were underutilized. Use of SGLT2 inhibitors was limited and ARNI therapy was not used. Digitalis was also not prescribed, which is in line with current thinking but not necessarily justified in patients with HF and reduced ejection fraction [21].

Nevertheless, a quadruple combination therapy, consisting of an AT-blocker, a beta-blocker, a mineralocorticoid receptor antagonist (MRA), and an SGLT2 inhibitor, which is known to improve prognosis, reduce hospitalizations, and lower mortality in acquired heart disease, received only 30 patients [22].

Given the substantial structural disease burden and the proportion of symptomatic patients, there appears to be a gap between available therapies and their real-world application.

Potential explanations include limited evidence in ACHD, particularly syndromic ACHD, as well as ACHD-specific feasibility barriers in selected physiologies, such as cyanosis/pulmonary hypertension, systemic right ventricle, or univentricular/Fontan circulation. In this context, reduced blood pressure and end-organ vulnerability (renal/hepatic) may limit tolerability. Additionally, concerns exist about adverse effects, drug–drug interactions, and adherence. Moreover, strong guideline recommendations for these subgroups are limited because ACHD is frequently an exclusion criterion in pivotal HF trials. These data highlight the need for prospective studies assessing safety, efficacy, tolerability, adherence, and drug–drug interactions of contemporary HF therapies in syndromic CHD.

Regarding pulmonary arterial hypertension therapy, targeted agents, including PDE5 inhibitors, endothelin receptor antagonists, and prostacyclin analogues, were administered as mono- or combination therapy. Sotatercept was not used in this cohort. Whether PAH therapies are equally effective and safe in syndromic ACHD compared with non-syndromic ACHD or idiopathic PAH cohorts remains uncertain. Systematic longitudinal analyses and ideally multicenter studies are needed to develop syndrome- and CHD-specific suggests strategies.

Oral anticoagulation was used in 20/107 patients (18.7%); 14 received vitamin K antagonists, 3 direct oral anticoagulants (DOAC), and 4/107 antiplatelet agents. This is clinically relevant, as use of DOACs in complex ACHD remains a debated area. Data on the use of DOACs are still limited particularly in complex ACHD, but real-world studies show promising safety and effectiveness. As some larger analyses suggest higher risks for bleeding/thrombosis compared with VKAs in this specific population, there is a need for careful patient selection and monitoring, particularly in complex CHD or cyanotic CHD or after Fontan operation [23,24,25].

Forty-one patients of 107 (38.3%) received thyroid hormone replacement, and 6 (5.6%) received oral iron supplementation (Table 6). This fact underscores the importance of multidisciplinary treatment of affected individuals, which goes far beyond purely cardiological aspects [19].

## 5. Limitations

This study has several limitations. First, the analysis is based on a relatively small subgroup, which limits statistical power and the robustness of conclusions, particularly for rare syndromes. While this registry represents a large real-world ACHD cohort, the number of patients with genetic syndromes/disorders (n = 107; 5.4%) limits statistical power for subgroup analyses and precludes syndrome-specific inferences for rare conditions. Accordingly, results should be interpreted as exploratory and hypothesis-generating, and non-significant findings may reflect limited power.

Second, the cohort is genetically and clinically heterogeneous, and registry-level data do not allow syndrome-specific deep dives for all entities. Third, a selection bias cannot be excluded, as patients were predominantly recruited from a specialist ACHD outpatient clinic, and severely impaired individuals with limited access to specialized care may be underrepresented.

Fourth, as the Pathfinder is a real-world registry, biomarker testing (including BNP/NT-proBNP) and imaging parameters were obtained indication-specific during routine care and were not mandated or uniformly available across centers. Therefore, no biomarker- or imaging-based numeric thresholds were used as required criteria for HF stage assignment in this baseline analysis.

Finally, the cross-sectional design precludes causal inference regarding the impact of individual comorbidities, prior interventions, or extracardiac disease on long-term outcomes.

Despite these limitations, our data provide an important first overview of structural characteristics, comorbidities, and contemporary management in syndromic ACHD with heart failure. They illustrate that improved survival comes with substantial multimorbidity and potentially underutilized therapeutic options. Future studies should refine syndrome-specific risk profiles, strengthen the evidence base for contemporary heart failure therapies in this population, and develop interdisciplinary care models tailored to the needs of syndromic ACHD patients.

## 6. Conclusions

Overall, this analysis depicts an aging, multimorbid, and interdisciplinary challenging patient population. Syndromic ACHD combine structurally complex and frequently surgically treated congenital heart disease with a high burden of cardiac comorbidities, such as aortopathy, pulmonary arterial hypertension, arrhythmias, and prior infective endocarditis, as well as extracardiac comorbidities involving endocrine, renal, and mental health domains.

These findings have clear implications for clinical care. First, affected individuals require lifelong follow-up in specialized ACHD centers with close integration of clinical genetics, endocrinology, nephrology, psychiatry/psychology, social medicine, and other disciplines. Second, structured screening programs for aortopathy, pulmonary arterial hypertension, thyroid disease, renal dysfunction, and mental health disorders should be implemented and systematically evaluated to enable early identification of high-risk individuals and targeted interventions. Finally, preventive strategies, including rehabilitation and prehabilitation, when appropriate, should be considered integral components of lifelong care for this special population.

## Figures and Tables

**Table 1:** Demographics of study population.

Characteristic	Overall N = 107	Male N = 42	Female N = 65	*p*-Value
Age (years)	33.4 ± 8.8 (34.0; 18.0–68.0)	33.4 ± 8.0 (34.0; 18.0–51.0)	33.5 ± 9.4 (33.0; 18.0–68.0)	0.861
Height (cm)	157.3 ± 10.3 (157.0; 126.0–178.0)	163.6 ± 8.0 (163.5; 150.0–178.0)	153.1 ± 9.5 (153.0; 126.0–174.0)	<0.001
Weight (kg)	67.9 ± 16.1 (67.0; 39.0–123.0)	71.3 ± 15.7 (71.0; 45.0–123.0)	65.7 ± 16.0 (63.5; 39.0–111.0)	0.070
BMI (kg/m^2^)	27.6 ± 6.8 (25.9; 15.2–52.8)	26.8 ± 6.3 (25.4; 17.1–42.3)	28.1 ± 7.2 (26.3; 15.2–52.8)	0.364
Body surface area (m^2^)	1.7 ± 0.2 (1.7; 1.2–2.3)	1.8 ± 0.2 (1.8; 1.4–2.3)	1.6 ± 0.2 (1.6; 1.2–2.0)	0.002

Mean ± SD (Median, range: Min–Max).

**Table 2:** Underlying leading congenital heart anomaly.

Characteristic	Overall N = 107	Trisomy 21 N = 49	Microdeletion 22q11.2 N = 27	Noonan N = 4	Turner N = 4	Other N = 23
Atrioventricular septal defect	42 (39%)	40 (82%)	0 (0%)	0 (0%)	0 (0%)	2 (8.7%)
Tetralogy of Fallot	19 (17%)	6 (12%)	9 (33%)	0 (0%)	0 (0%)	4 (17%)
Pulmonary atresia with ventricular septal defect	7 (6.4%)	0 (0%)	6 (22%)	0 (0%)	0 (0%)	1 (4.3%)
Truncus arteriosus communis	6 (5.5%)	0 (0%)	5 (19%)	0 (0%)	0 (0%)	1 (4.3%)
Ventricular septal defect	6 (5.5%)	3 (6.1%)	2 (7.4%)	0 (0%)	0 (0%)	1 (4.3%)
Aortic valve disease	4 (3.6%)	0 (0%)	0 (0%)	0 (0%)	1 (25%)	3 (13%)
Interrupted aortic arch	4 (3.7%)	0 (0%)	4 (15%)	0 (0%)	0 (0%)	0 (0%)
Double outlet right ventricle	4 (3.7%)	0 (0%)	1 (3.7%)	0 (0%)	0 (0%)	3 (13%)
Congenitally corrected transposition of great arteries	3 (2.8%)	0 (0%)	0 (0%)	0 (0%)	0 (0%)	3 (13%)
Aortic coarctation	3 (2.8%)	0 (0%)	0 (0%)	0 (0%)	3 (75%)	0 (0%)
Aortic stenosis, subvalvular	1 (0.9%)	0 (0%)	0 (0%)	1 (17%)	0 (0%)	0 (0%)
Aortic stenosis, supravalvular	1 (0.9%)	0 (0%)	0 (0%)	0 (0%)	0 (0%)	1 (4.3%)
Atrial septal defect	1 (0.9%)	0 (0%)	0 (0%)	1 (17%)	0 (0%)	0 (0%)
Pulmonary stenosis	1 (0.9%)	0 (0%)	0 (0%)	1 (17%)	0 (0%)	0 (0%)
Transposition of great arteries	1 (0.9%)	0 (0%)	0 (0%)	1 (17%)	0 (0%)	0 (0%)
Univentricular heart	2 (1.8%)	0 (0%)	0 (0%)	0 (0%)	0 (0%)	2 (8.7%)
Mitral stenosis, congenital	1 (0.9%)	0 (0%)	0 (0%)	0 (0%)	0 (0%)	1 (4.3%)
Marfan Syndrome	1 (0.9%)	0 (0%)	0 (0%)	0 (0%)	0 (0%)	1 (4.3%)

**Table 3:** Overview of anatomical, functional, and treatment characteristics in syndromic adults with congenital heart disease.

Characteristic	Overall N = 107	Trisomy 21 N = 49	Microdeletion 22q11.2 N = 27	Noonan N = 4	Turner N = 4	Other N = 23
**Systemic ventricular morphology**						
Right	3 (2.8%)	0 (0%)	0 (0%)	0 (0%)	0 (0%)	3 (13%)
Left	102 (95%)	49 (100%)	27 (100%)	4 (100%)	4 (100%)	18 (78%)
Univentricular	2 (1.9%)	0 (0%)	0 (0%)	0 (0%)	0 (0%)	2 (8.7%)
**Cyanotic state**						
Acyanotic	67 (63%)	42 (86%)	6 (22%)	3 (75%)	4 (100%)	12 (52%)
Primary cyanosis	40 (37%)	7 (14%)	21 (78%)	1 (25%)	0 (0%)	11 (48%)
Secondary cyanosis	7 (6.5%)	7 (14%)	0 (0%)	0 (0%)	0 (0%)	0 (0%)
**Treatment status**						
Native	8 (7.5%)	5 (10%)	0 (0%)	0 (0%)	0 (0%)	3 (13%)
Operative palliation	3 (2.8%)	1 (2.0%)	1 (3.7%)	0 (0%)	0 (0%)	1 (4.3%)
Operative repair	71 (66%)	42 (86%)	12 (44%)	2 (50%)	1 (25%)	14 (61%)
Interventional	25 (23%)	1 (2.0%)	14 (52%)	2 (50%)	3 (75%)	5 (22%)

*Data are presented as n (%).*

**Table 4:** Overview of heart failure classification according to CHD-adapted ACC/AHA staging system and Perloff functional class.

Characteristic	Overall N = 107	Trisomy 21 N = 49	Microdeletion 22q11.2 N = 27	Noonan N = 4	Turner N = 4	Other N = 23
**ACC/AHA CHD-HF Classification**						
B	30 (28%)	13 (27%)	4 (15%)	1 (25%)	4 (100%)	8 (35%)
C	75 (70%)	34 (69%)	23 (85%)	3 (75%)	0 (0%)	15 (65%)
D	2 (1.9%)	2 (4.1%)	0 (0%)	0 (0%)	0 (0%)	0 (0%)
**Perloff functional class**						
I–II	97 (91%)	45 (92%)	25 (93%)	3 (75%)	4 (100%)	20 (87%)
III	9 (8.4%)	3 (6.1%)	2 (7.4%)	1 (25%)	0 (0%)	3 (13%)
IV	1 (0.9%)	1 (2.0%)	0 (0%)	0 (0%)	0 (0%)	0 (0%)

*Data are presented as n (%).*

**Table 5:** Overview of cardiovascular and non-cardiac comorbidities in syndromic adults with congenital heart disease and heart failure, including sex-specific distributions.

Characteristic	Overall N = 107	Trisomy 21 N = 49	Microdeletion 22q11.2 N = 27	Noonan N = 4	Turner N = 4	Other N = 23
**Cardiac comorbidities**						
Aortopathy	49 (46%)	8 (16%)	23 (85%)	1 (25%)	4 (100%)	13 (57%)
Pulmonary hypertension	12 (11%)	8 (16%)	0 (0%)	1 (25%)	0 (0%)	3 (13%)
Arrhythmias	10 (9.3%)	2 (4.1%)	2 (7.4%)	0 (0%)	0 (0%)	6 (26%)
Infective endocarditis	9 (8.4%)	1 (2.0%)	4 (15%)	0 (0%)	0 (0%)	4 (17%)
Arterial hypertension	6 (5.6%)	3 (6.1%)	1 (3.7%)	0 (0%)	0 (0%)	2 (8.7%)
Cyanosis	7 (6.5%)	3 (6.1%)	2 (7.4%)	0 (0%)	0 (0%)	2 (8.7%)
**Non-cardiac comorbidities**						
Iron deficiency	4 (3.7%)	1 (2.0%)	0 (0%)	1 (25%)	1 (25%)	1 (4.3%)
Liver failure	6 (5.6%)	4 (8.2%)	1 (3.7%)	0 (0%)	0 (0%)	1 (4.3%)
Kidney disease	16 (15%)	6 (12%)	5 (19%)	0 (0%)	1 (25%)	4 (17%)
Hyperuricemia	13 (12%)	10 (20%)	0 (0%)	1 (25%)	0 (0%)	2 (8.7%)
Neurological disorders	10 (9.3%)	3 (6.1%)	1 (3.7%)	0 (0%)	0 (0%)	6 (26%)
Sleep apnea syndrome	2 (1.9%)	2 (4.1%)	0 (0%)	0 (0%)	0 (0%)	0 (0%)
Diabetes mellitus	1 (0.9%)	0 (0%)	1 (3.7%)	0 (0%)	0 (0%)	0 (0%)
Hyperlipidemia	5 (4.7%)	1 (2.0%)	2 (7.4%)	0 (0%)	0 (0%)	2 (8.7%)
Lp(a) elevation	6 (5.6%)	1 (2.0%)	1 (3.7%)	2 (50%)	0 (0%)	2 (8.7%)
Hypothyroidism	26 (24%)	18 (37%)	5 (19%)	1 (25%)	0 (0%)	2 (8.7%)
Hyperthyroidism	2 (1.9%)	1 (2.0%)	1 (3.7%)	0 (0%)	0 (0%)	0 (0%)
Thyroiditis	6 (5.6%)	4 (8.2%)	0 (0%)	0 (0%)	2 (50%)	0 (0%)
Depression	15 (14%)	5 (10%)	6 (22%)	1 (25%)	0 (0%)	3 (13%)

*Data are presented as n (%).*

**Table 6:** Overview of cardiovascular and general medication use in syndromic adults with congenital heart disease.

Characteristic	Overall N = 107	Trisomy 21 N = 49	Microdeletion 22q11.2 N = 27	Noonan N = 4	Turner N = 4	Other N = 23
No medication	41 (38%)	16 (33%)	13 (48%)	1 (25%)	1 (25%)	10 (43%)
**Cardiac medication**						
Beta-blocker	25 (23%)	5 (10%)	6 (22%)	3 (75%)	2 (50%)	9 (39%)
Angiotensin-converting enzyme (ACE) inhibitors	3 (2.8%)	2 (4.1%)	0 (0%)	0 (0%)	0 (0%)	1 (4.3%)
Angiotensin II receptor blockers	6 (5.6%)	1 (2.0%)	1 (3.7%)	0 (0%)	1 (25%)	3 (13%)
Calcium channel blocker	1 (0.9%)	0 (0%)	0 (0%)	0 (0%)	0 (0%)	1 (4.3%)
Diuretics	10 (9.3%)	5 (10%)	3 (11%)	1 (25%)	0 (0%)	1 (4.3%)
Mineralocorticoid receptor antagonists	8 (7.5%)	3 (6.1%)	3 (11%)	1 (25%)	0 (0%)	1 (4.3%)
SGLT2 inhibitors	3 (2.8%)	1 (2.0%)	1 (3.7%)	0 (0%)	0 (0%)	1 (4.3%)
Antiarrhythmic agents	1 (0.9%)	0 (0%)	0 (0%)	0 (0%)	1 (25%)	0 (0%)
“Fantastic four”	30 (28%)	7 (14%)	7 (26%)	3 (75%)	3 (75%)	10 (43%)
**Targeted PAH medication**						
PDE-5 inhibitor	7 (6.5%)	4 (8.2%)	1 (3.7%)	1 (25%)	0 (0%)	1 (4.3%)
Endothelin receptor antagonist (ERA)	6 (5.6%)	3 (6.1%)	0 (0%)	1 (25%)	0 (0%)	2 (8.7%)
Prostanoids	1 (0.9%)	0 (0%)	0 (0%)	1 (25%)	0 (0%)	0 (0%)
PAH others	3 (2.8%)	2 (4.1%)	0 (0%)	0 (0%)	1 (25%)	0 (0%)
**Non-cardiac medication**						
Vitamin K antagonist	14 (13%)	6 (12%)	2 (7.4%)	0 (0%)	1 (25%)	5 (22%)
Direct oral anticoagulants (DOACs)	3 (2.8%)	1 (2.0%)	1 (3.7%)	1 (25%)	0 (0%)	0 (0%)
Acetylsalicylic acid (ASS)	3 (2.8%)	0 (0%)	2 (7.4%)	1 (25%)	0 (0%)	0 (0%)
Clopidogrel	1 (0.9%)	0 (0%)	1 (3.7%)	0 (0%)	0 (0%)	0 (0%)
Thyroid medication	41 (38%)	27 (55%)	8 (30%)	1 (25%)	2 (50%)	3 (13%)
Statins	1 (0.9%)	0 (0%)	0 (0%)	0 (0%)	0 (0%)	1 (4.3%)
Iron supplementation, orally	6 (5.6%)	3 (6.1%)	1 (3.7%)	0 (0%)	1 (25%)	1 (4.3%)

## Data Availability

The original contributions presented in this study are included in the article. Further inquiries can be directed to the corresponding author.
